# Therapeutic effects of mitoquinol during an acute heat stress challenge in growing barrows

**DOI:** 10.1093/jas/skae161

**Published:** 2024-06-11

**Authors:** Edith J Mayorga, Erin A Horst, Brady M Goetz, Sonia Rodriguez-Jimenez, Megan A Abeyta, Mohmmad Al-Qaisi, Robert P Rhoads, Joshua T Selsby, Lance H Baumgard

**Affiliations:** Department of Animal Science, Iowa State University, Ames, IA 50011, USA; Department of Animal Science, Iowa State University, Ames, IA 50011, USA; Department of Animal Science, Iowa State University, Ames, IA 50011, USA; Department of Animal Science, Iowa State University, Ames, IA 50011, USA; Department of Animal Science, Iowa State University, Ames, IA 50011, USA; Department of Animal Science, Iowa State University, Ames, IA 50011, USA; Department of Animal and Poultry Sciences, Virginia Tech, Blacksburg, VA 24061, USA; Department of Animal Science, Iowa State University, Ames, IA 50011, USA; Department of Animal Science, Iowa State University, Ames, IA 50011, USA

**Keywords:** antioxidant, heat stroke, hyperthermia, inflammation, MitoQ

## Abstract

Study objectives were to determine the effects of mitoquinol (**MitoQ**, a mitochondrial-targeted antioxidant) on biomarkers of metabolism and inflammation during acute heat stress (**HS**). Crossbred barrows [*n* = 32; 59.0 ± 5.6 kg body weight (**BW)**] were blocked by BW and randomly assigned to 1 of 4 environmental-therapeutic treatments: 1) thermoneutral (**TN**) control (*n* = 8; **TNCon**), 2) TN and MitoQ (*n* = 8; **TNMitoQ**), 3) HS control (*n* = 8; **HSCon**), or 4) HS and MitoQ (*n* = 8; **HSMitoQ)**. Pigs were acclimated for 6 d to individual pens before study initiation. The trial consisted of two experimental periods (**P**). During P1 (2 d), pigs were fed ad libitum and housed in TN conditions (20.6 ± 0.8 °C). During P2 (24 h), HSCon and HSMitoQ pigs were exposed to continuous HS (35.2 ± 0.2 °C), while TNCon and TNMitoQ remained in TN conditions. MitoQ (40 mg/d) was orally administered twice daily (0700 and 1800 hours) during P1 and P2. Pigs exposed to HS had increased rectal temperature, skin temperature, and respiration rate (+1.5 °C, +6.8 °C, and +101 breaths per minute, respectively; *P* < 0.01) compared to their TN counterparts. Acute HS markedly decreased feed intake (**FI**; 67%; *P* < 0.01); however, FI tended to be increased in HSMitoQ relative to HSCon pigs (1.5 kg vs. 0.9 kg, respectively; *P* = 0.08). Heat-stressed pigs lost BW compared to their TN counterparts (−4.7 kg vs. +1.6 kg, respectively; *P* < 0.01); however, the reduction in BW was attenuated in HSMitoQ compared to HSCon pigs (−3.9 kg vs. −5.5 kg, respectively; *P* < 0.01). Total gastrointestinal tract weight (empty tissue and luminal contents) was decreased in HS pigs relative to their TN counterparts (6.2 kg vs. 8.6 kg, respectively; *P* < 0.01). Blood glucose increased in HSMitoQ relative to HSCon pigs (15%; *P* = 0.04). Circulating non-esterified fatty acids (**NEFA**) increased in HS compared to TN pigs (*P* < 0.01), although this difference was disproportionately influenced by elevated NEFA in HSCon relative to HSMitoQ pigs (251 μEq/L vs. 142 μEq/L; *P* < 0.01). Heat-stressed pigs had decreased circulating insulin relative to their TN counterparts (47%; *P* = 0.04); however, the insulin:FI ratio tended to increase in HS relative to TN pigs (*P* = 0.09). Overall, circulating leukocytes were similar across treatments (*P* > 0.10). Plasma C-reactive protein remained similar among treatments; however, haptoglobin increased in HS relative to TN pigs (48%; *P* = 0.03). In conclusion, acute HS exposure negatively altered animal performance, inflammation, and metabolism, which were partially ameliorated by MitoQ.

## Teaser

Intestinal ischemia, increased reactive oxygen species production, and subsequent oxidative stress might contribute to intestinal barrier dysfunction during heat stress. This study demonstrated that mitoquinol, a mitochondria-targeted antioxidant, positively influenced growth performance during an acute heat stress event in growing pigs but had little impact on metabolism and systemic inflammation.

## Introduction

Environmental hyperthermia negatively impacts every sector of animal agriculture and causes enormous financial losses annually (St-Pierre et al., 2003). This lost revenue is mainly associated with reduced animal productivity as heat stress (**HS**) adversely impacts growth rate, production efficiency, and reproductive performance (Baumgard and Rhoads, 2013; Mayorga et al., 2020). While the cause of reduced animal productivity during HS is likely multifactorial, increasing evidence suggests the etiology of these undesirable effects stems, at least in part, from a compromised gastrointestinal tract (**GIT**) barrier (Mayorga et al., 2020). The exact pathophysiological mechanism(s) responsible for HS-induced intestinal hyperpermeability is still being elucidated (Mayorga et al., 2020), but a reprioritization of blood trafficking has traditionally been an ostensible culprit (Hall et al., 1999, 2001). During HS, thermoregulatory mechanisms are implemented to maximize radiant heat dissipation. Consequently, blood flow is redistributed towards the periphery and away from the splanchnic bed; a circulatory process that reduces blood delivery to the GIT (Kregel et al., 1988; Hall et al., 2001). These events culminate in the production of reactive oxygen species (**ROS**) and subsequent oxidative stress. Oxidative damage to epithelial cells compromises intestinal barrier integrity, allowing the translocation of bacterial contents (i.e., lipopolysaccharide [**LPS**]) into circulation, contributing to local and systemic inflammatory responses (Lambert, 2004, 2009; Burhans et al., 2022).

Gastrointestinal oxidative stress has been previously reported in heat-stressed pigs (Pearce et al., 2013; Liu et al., 2016), rodents (Yu et al., 2011; Cheng et al., 2019a), and poultry (Cheng et al., 2019b;). In particular, HS reduces antioxidant capacity and increases intestinal tissue ROS (Oliver et al., 2012; Pearce et al., 2013; Liu et al., 2016). Additionally, intestinal epithelial membrane damage and inflammation biomarkers increase during a heat load, likely as a consequence of oxidation-induced intestinal injury (Oliver et al., 2012). Thus, intestinal oxidative stress appears to be a key component of HS-induced intestinal hyperpermeability.

Considering oxidative stress’ presumed etiological role in HS-induced intestinal damage, multiple efforts have been made to attenuate it (Maseko et al., 2014; Liu et al., 2016, 2018a, b). However, in vivo responses to dietary antioxidants during HS are inconsistent, as some have been successful at reducing oxidative stress markers (Maseko et al., 2014; Liu et al., 2016; Cheng et al., 2019b), while others have reported little or no benefits (Liu et al., 2018a, b; Silva-Guillen et al., 2019). Further, antioxidant supplementation during HS has previously been shown to alleviate the impact of HS on performance (Chauhan et al., 2014; Lv et al., 2015), but not in others (Shakeri et al., 2019; Silva-Guillen et al., 2019; Abeyta et al., 2023). Thus, there remains a need to develop a more effective and reliable strategy to mitigate oxidative stress and the ensuing intestinal inflammation (and ultimately animal performance) during a heat load.

Mitochondria constitute the main source of endogenous ROS (Turrens, 2003), making them particularly vulnerable to oxidative damage; thus, mitochondria represent a logical target for applying therapeutic strategies aimed at reducing oxidative stress. Mitoquinol (**MitoQ**; C_37_H_46_O_4_P^+^) is an orally available antioxidant that targets mitochondria (Murphy and Smith, 2007). Previous disease models have successfully assessed the role of MitoQ in reducing mitochondrial damage (Adlam et al., 2005; Supinski et al., 2009) and protecting tissue injury during sepsis (Lowes et al., 2008; Zhang et al., 2020). Therefore, we hypothesized that MitoQ administration during a heat load would reduce inflammation and alter metabolism. Hence, the current study evaluated the therapeutic effects of MitoQ on metabolism and inflammatory biomarkers during an acute HS challenge in growing pigs.

## Materials and Methods

All experimental procedures followed the guidelines for the ethical and humane use of animals for research according to the Guide for the Care and Use of Agricultural Animals in Research and Teaching (FASS, 2010) and were approved by the Iowa State University Institutional Animal Care and Use Committee (#18-314). Data about HS-mediated changes in skeletal muscle from these pigs have been previously reported (Rudolph et al., 2021).

### Animals, housing, and experimental design

Thirty-two crossbred barrows [59.0 ± 5.6 kg body weight (**BW)**] were utilized in an experiment conducted at the Iowa State University Swine Nutrition Farm facility (Ames, IA). Based on BW, pigs were allocated to 8 blocks and randomly assigned to 1 of 4 environmental-therapeutic treatments: 1) thermoneutral (**TN**) control (*n *= 8; **TNCon**), 2) TN and MitoQ (*n* = 8; **TNMitoQ**), 3) HS control (*n* = 8; **HSCon**), or 4) HS and MitoQ (*n* = 8; **HSMitoQ**). Pigs were housed in individual crates (57 × 221 cm) equipped with a stainless-steel feeder and a nipple drinker. Pigs were manually fed a standard diet formulated to meet or exceed the essential amino acid, mineral, and vitamin requirements for growing pigs (NRC, 2012; Table 1). Feed and water were provided ad libitum during the entire experiment.

**Table 1.** Ingredient composition of diet (as-fed basis)

Ingredient	%
Corn	63.99
Soybean meal, CP 46%	13.67
Corn DDGS^1^	20.21
Lysine HCl	0.29
Limestone	1.26
NaCl	0.43
Vitamin–mineral premix^2^	0.13
Phytase (500 FTU/kg)^3^	0.02

^1^Corn distillers dried grains with solubles.

^2^Vitamin–mineral premix provided the following (per kilogram diet): 8,400 IU/kg of vitamin A, 1,540 IU/kg of vitamin D_3_, 45 IU of vitamin E, 0.03 mg of vitamin B_12_, 2.2 mg of menadione, 4.2 mg of riboflavin, 17 mg of d-pantothenic acid, 21 mg of niacin, 1.9 mg of ethoxyquin, 112 mg of Fe (ferrous sulfate), 112 mg of Zn (zinc sulfate), 51 mg of Mn (manganous oxide), 20 mg of Cu (copper chloride), 0.78 mg of I (calcium iodate), and 0.17 mg of Se (sodium selenite).

^3^Ronozyme (DSM Nutritional Products Ltd, Basel, Switzerland).

Following 6 d of acclimation to individual pens, pigs were enrolled in two experimental periods (**P**). During P1 (2 d), pigs were housed in TN conditions (20.6 ± 0.8 °C; 60.0 ± 0.5% relative humidity) for the collection of baseline body temperature indices and production parameters. During P2 (24 h), HSCon and HSMitoQ pigs were exposed to constant HS (35.2 ± 0.2 °C; 29.2 ± 0.3% relative humidity), while TNCon and TNMitoQ remained in TN conditions. Room temperature and humidity were monitored and recorded every 5 min by a data logger (Lascar EL-USB-2LCD, Erie, PA).

Therapeutic treatments consisted of 10 g of cookie dough with no therapeutic enrichment (**Con**) or 20 mg MitoQ (40 mg/d; MitoQ, Auckland, New Zealand). Treatments were administered orally twice daily (~0730 and 1830 hours) during P1 and P2. The MitoQ dose regimen was adapted from previous experiments in rodents and humans (Supinski et al., 2009; Snow et al., 2010).

### Body temperature measurements

Rectal temperature (*T*_R_), skin temperature (*T*_S_), and respiration rate (**RR**) were obtained twice daily (~0700 and 1800 hours) during P1, and at 0, 4, 8, 12, 16, 20, and 24 h during P2. Rectal temperature was measured using an electronic thermometer (SureTemp Plus 590, accuracy: ±0.1 °C WelchAllyn, Skaneateles Falls, NY). Skin temperature was measured at the rump area using a calibrated infrared thermometer (IRT207: The Heat Seeker 8:1 Mid-Range Infrared Thermometer, accuracy: ±2 °C; General Tools, New York, NY), and RR was determined by counting flank movements for 15 s and multiplied by 4 to obtain breaths per minute (**bpm**).

### Production parameters

Feed intake (**FI**) was measured daily during P1 and at 4, 8, 12, 16, 20, and 24 h during P2 as feed disappearance. BW were recorded at the end of the acclimation period, at the beginning of P2, and immediately before sacrifice. BW change was calculated by subtracting the final BW from the BW recorded before the initiation of the environmental challenge.

### Organ weights and ileum tissue collection

Pigs were sacrificed at the end of the 24 h challenge with the captive bolt technique followed by exsanguination. The stomach and small and large intestines were removed, and their contents weighed. Weights of the empty stomach and small and large intestines were also recorded to determine total GIT weight.

### Blood sampling and analysis

Blood samples (plasma; K_2_EDTA vacutainers, Franklin Lakes, NJ) were obtained during exsanguination. Plasma samples were harvested by centrifugation at 1,500 × *g* for 15 min at 4 °C, aliquoted, and stored at −20 °C until analysis. A second set of blood samples was submitted to the Iowa State Department of Veterinary Pathology (Ames, IA) for automated differential complete cell blood count using a flow cytometry-based hematology analyzer (ADVIA 2120i; Siemens, Munich, Germany). Plasma glucose, non-esterified fatty acids (**NEFA**), blood urea nitrogen (**BUN**), insulin, C-reactive protein (**C-RP**), and haptoglobin (**Hp**) concentrations were measured using commercially available kits following the manufacturer’s instructions (glucose and NEFA, Wako Chemicals USA, Inc., Richmond, VA; BUN, Teco Diagnostics, Anaheim, CA; insulin, Mercodia Porcine Insulin ELISA, Mercodia AB, Uppsala, Sweden; C-RP and Hp, Life Diagnostics Inc., West Chester, PA). The intra-assay coefficients of variation for glucose, insulin, C-RP, and Hp were 3.6%, 4.2%, 2.8%, and 1.9%, respectively. The intra- and inter-assay coefficients of variation for NEFA and BUN were 3.7% and 2.7%, and 4.1% and 2.7%, respectively.

### Statistical analysis

All data were statistically analyzed using the MIXED procedure of SAS version 9.4 (SAS Inst. Inc., Cary, NC). Hourly measurements (i.e., body temperature indices and FI) were analyzed by repeated measures with an autoregressive covariance structure and time as the repeated factor. For body temperature indices, each variable’s time 0 served as a covariate in the model. The model included treatment, time, treatment × time interaction, and block as fixed effects; pig was used as the random effect. Additionally, blood parameters, BW, and GIT weights were analyzed using PROC MIXED with a diagonal covariance structure. The model included treatment as a fixed effect. Pre-planned contrasts were assessed to evaluate environmental (i.e., TN vs. HS) and therapeutic (i.e., Con vs. MitoQ) effects. Data are reported as least squares means and considered significant if *P* ≤ 0.05 and a tendency if 0.05 < *P* ≤ 0.10.

## Results

### Body temperature indices and growth performance

As expected, HS pigs had increased *T*_R_, *T*_S_, and RR compared to their TN counterparts (1.5 °C, 6.8 °C, and 101 bpm, respectively; *P* < 0.01; Table 2). Although there were no differences in *T*_R_ or *T*_S_ due to MitoQ administration, RR was decreased in HSMitoQ compared to HSCon pigs (9 bpm; *P* = 0.03).

**Table 2.** Effects of mitoquinol (MitoQ) on body temperature indices, FI, and GIT measurements during a 24-h acute HS challenge in growing pigs

Parameter	Treatment^1^				SEM	*P* value			Contrasts^4^		
	TNCo	TNMitoQ	HSCon	HSMitoQ		Trt^2^	Time	Trt × Time^3^	TN vs. HS	HSCon vs. HSMitoQ	Con vs. MitoQ
Body temp. indices	*n*										
*T*_R_^5^, °C	39.11^b^	39.05^b^	40.59^a^	40.49^a^	0.05	<0.01	<0.01	0.02	<0.01	0.20	0.16
*T*_S_^6^, °C	34.90^b^	35.04^b^	41.79^a^	41.73^a^	0.18	<0.01	<0.01	<0.01	<0.01	0.80	0.85
RR^7^, bpm	36^c^	34^c^	140^a^	131^b^	3	<0.01	0.43	0.38	<0.01	0.03	0.07
FI^8^, kg	3.55^a^	3.84^a^	0.94^b^	1.52^b^	0.23	<0.01	—	—	<0.01	0.08	0.06
GIT weight^9^, kg	8.64^a^	8.58^a^	5.89^b^	6.47^b^	0.24	<0.01	—	—	<0.01	0.10	0.29
Empty GIT^10^, kg	4.18^a^	4.22^a^	3.43^b^	3.38^b^	0.07	<0.01	—	—	<0.01	0.60	0.93
Luminal cont.^11^, kg	4.46^a^	4.36^a^	2.46^c^	3.09^b^	0.20	<0.01	—	—	<0.01	0.04	0.21
Luminal cont.^12^, %	128	115	277	236	24	<0.01	—	—	<0.01	0.25	0.28

^1^TNCon = thermoneutral control; TNMitoQ = thermoneutral + MitoQ; HSCon = heat stress control; HSMitoQ = heat stress + MitoQ.

^2^Treatment.

^3^Treatment by time interaction.

^4^Contrasts statements: TN = TNCon + TNMitoQ; HS = HSCon + HSMitoQ; Con = TNCon + HSCon; MitoQ = TNMitoQ + HSMitoQ.

^5^Rectal temperature.

^6^Skin temperature.

^7^Respiration rate.

^8^Feed intake.

^9^Total gastrointestinal tract weight (tissue + luminal contents from stomach and small and large intestines).

^10^Empty gastrointestinal weight (stomach and small and large intestines).

^11^Gastrointestinal luminal contents (from stomach and small and large intestines).

^12^Luminal contents as a percentage of FI.

^a,b,c^Means within a row with different superscripts significantly differ (*P *≤ 0.05).

**Figure 1.**
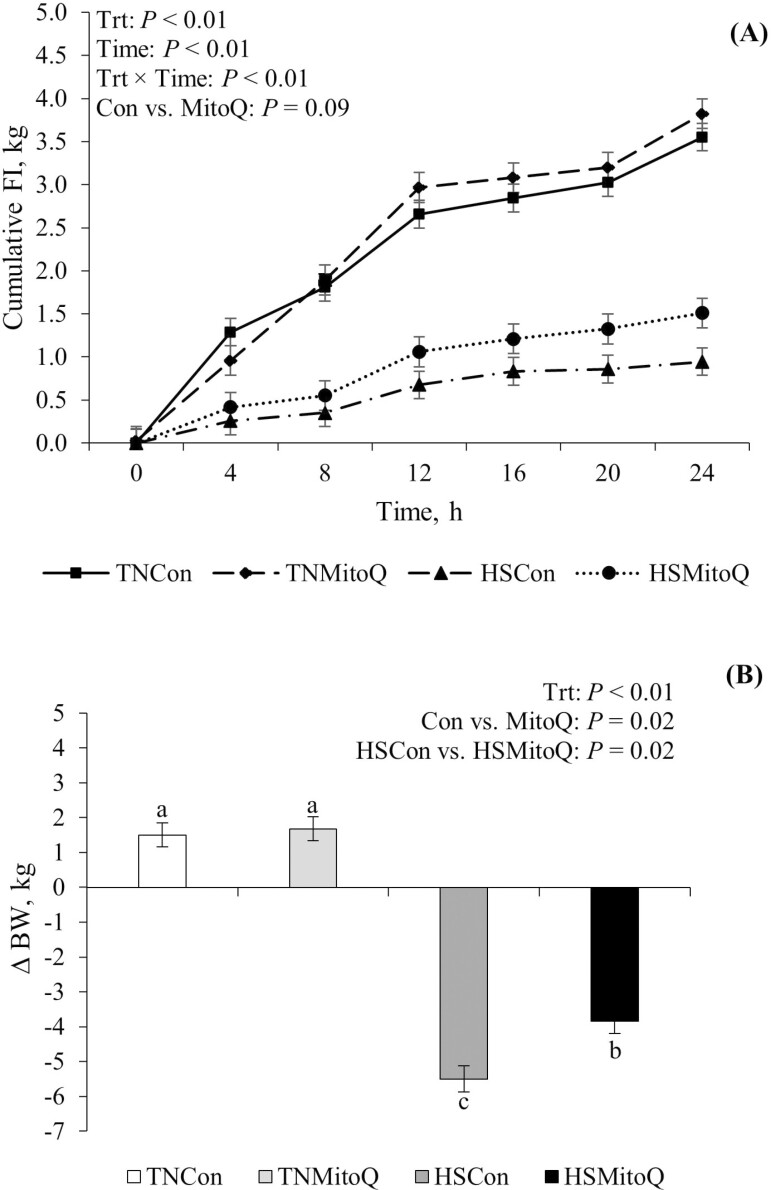
Effects of mitoquinol (**MitoQ**) on (A) cumulative FI and (B) delta BW (final—initial BW) during a 24 h acute HS challenge in growing pigs. Treatments: TNCon = Thermoneutral control; TNMitoQ = Thermoneutral MitoQ; HSCon = Heat stress control; HSMitoQ = Heat stress MitoQ. Data are represented as least squares means ± standard error of the mean. ^a,b,c^Values with differing superscripts denote overall treatment differences (*P* ≤ 0.05).

Pigs exposed to HS had a marked decrease in FI compared to their TN counterparts (67%; *P* < 0.01; Table 2; Figure 1A). FI tended to be increased in HSMitoQ relative to HSCon pigs (1.5 kg vs. 0.9 kg, respectively; *P* = 0.08; Table 2). Additionally, an overall tendency for increased FI was observed in MitoQ compared to their Con counterparts (19%; *P* = 0.06; Table 2). By the end of the 24 h HS challenge, HS pigs lost BW while TN pigs gained BW (−4.7 kg vs. +1.6 kg, respectively; *P* < 0.01; Figure 1B). However, this reduction in BW was attenuated in HSMitoQ relative to HSCon pigs (−3.9 kg vs. −5.5 kg, respectively; *P* < 0.01; Figure 1B).

### Gastrointestinal tract weight

Total GIT weight decreased in HS relative to TN pigs (6.2 kg vs. 8.6 kg; *P* < 0.01; Table 2). This response was influenced by both reduced empty GIT weight (3.4 kg vs. 4.2 kg; *P* < 0.01) and decreased GIT contents (2.8 kg vs. 4.4 kg; *P* < 0.01) in HS pigs relative to their TN counterparts. No MitoQ effects were observed on total or empty GIT weight; however, GIT contents were increased in HSMitoQ pigs compared to their HSCon counterparts (3.1 kg vs. 2.5 kg; *P* = 0.04; Table 2). Total luminal contents as a percentage of FI increased in HS relative to TN pigs (256% vs. 121%; *P* < 0.01; Table 2).

### Blood hematology, and metabolic and inflammatory biomarkers

Overall, no treatment differences were observed on any hematologic parameter analyzed in the current study (*P* > 0.12; Table 3). In addition, plasma C-RP was not altered by either environmental or therapeutic treatment; however, circulating Hp was increased in HS pigs relative to their TN counterparts (48%; *P* = 0.03; Table 3). Metabolic responses to HS are presented in Table 4. Overall, plasma glucose tended to differ across treatments and was reduced in HSCon relative to TN and HSMitoQ pigs (14%; *P* = 0.07). This response also influenced an effect between HS treatments as circulating glucose increased in HSMitoQ relative to HSCon pigs (15%; *P* = 0.04). Although circulating insulin did not differ across treatments (*P* > 0.20), heat-stressed pigs had decreased insulin relative to their TN counterparts (47%; *P* = 0.04). However, the insulin:FI ratio tended to increase in HS relative to TN pigs (50%; *P* = 0.09). Circulating NEFA increased in HS relative to TN pigs; however, this difference was mainly driven by increased NEFA concentrations in HSCon pigs relative to TN and HSMitoQ pigs (251 μEq/L vs. 124 μEq/L; *P* < 0.01). BUN concentrations remained similar across treatments (*P* > 0.36).

**Table 3.** Effects of mitoquinol (MitoQ) on hematology and inflammatory biomarkers during a 24-h acute HS challenge in growing pigs

Parameter	Treatment^1^				SEM	*P* value	Contrasts^3^		
	TNCon	TNMitoQ	HSCon	HSMitoQ		Trt^2^	TN vs. HS	HSCon vs. HSMitoQ	Con vs. MitoQ
Hematology									
WBC^4^, ×10^3^/μL	22.30	25.30	21.97	23.28	3.00	0.83	0.69	0.74	0.55
Neutrophils, ×10^3^/μL	7.91	8.29	8.34	7.93	2.41	0.99	0.99	0.90	0.99
Lymph^5^., ×10^3^/μL	12.39	14.55	12.18	13.21	1.83	0.76	0.67	0.67	0.47
Monocytes, ×10^3^/μL	1.12	1.38	0.88	1.13	0.17	0.23	0.19	0.30	0.24
Eosinophils, ×10^3^/μL	0.54	0.54	0.36	0.49	0.13	0.65	0.38	0.46	0.67
Basophils, ×10^3^/μL	0.25	0.34	0.23	0.39	0.15	0.85	0.91	0.44	0.49
RBC^6^, × 10^6^/μL	7.57	6.97	7.70	7.98	0.49	0.41	0.26	0.67	0.78
Hemoglobin, g/dL	13.06	12.17	13.27	14.35	0.61	0.12	0.09	0.22	0.89
Hematocrit, %	40.10	37.88	41.19	44.11	2.90	0.40	0.23	0.46	0.92
Inflammation									
C-RP^7^, μg/mL	38.2	30.1	39.0	33.5	6.1	0.71	0.73	0.53	0.28
Hp^8^, μg/mL	266	311	435	421	61	0.17	0.03	0.88	0.80

^1^TNCon = thermoneutral control; TNMitoQ = thermoneutral + MitoQ; HSCon = heat stress control; HSMitoQ = heat stress + MitoQ.

^2^Treatment.

^3^Contrasts statements: TN = TNCon + TNMitoQ; HS = HSCon + HSMitoQ; Con = TNCon + HSCon; MitoQ = TNMitoQ + HSMitoQ.

^4^White blood cells.

^5^Lymphocytes.

^6^Red blood cells.

^7^C-reactive protein.

^8^Haptoglobin.

**Table 4.** Effects of mitoquinol (MitoQ) on metabolism during a 24-h acute HS challenge in growing pigs

Parameter	Treatment^1^				SEM	*P* value	Contrasts^3^		
	TNCon	TNMitoQ	HSCon	HSMitoQ		Trt^2^	TN vs. HS	HSCon vs. HSMitoQ	Con vs. MitoQ
Glucose, mg/dL	128^x^	121^xy^	107^y^	123^x^	5	0.07	0.11	0.04	0.40
Insulin, μg/L	0.16	0.16	0.08	0.09	0.03	0.20	0.04	0.74	0.84
Insulin:FI^4^	0.05	0.03	0.07	0.05	0.01	0.26	0.09	0.34	0.22
NEFA^5^, μEq/L	110^b^	120^b^	251^a^	142^b^	24	<0.01	<0.01	<0.01	0.05
BUN^6^, mg/dL	11.6	11.4	13.7	12.0	1.0	0.36	0.19	0.23	0.34

^1^TNCon = thermoneutral control; TNMitoQ = thermoneutral + MitoQ; HSCon = heat stress control; HSMitoQ = heat stress + MitoQ.

^2^Treatment.

^3^Contrasts statements: TN = TNCon + TNMitoQ; HS = HSCon + HSMitoQ; Con = TNCon + HSCon; MitoQ = TNMitoQ + HSMitoQ.

^4^Insulin-to-FI ratio.

^5^Non-esterified fatty acids.

^6^Blood urea nitrogen.

^a,b^Means with different superscripts denote an overall treatment difference (*P* ≤ 0.05).

^x,y^Means with different superscripts denote an overall treatment tendency (0.05 < *P* ≤ 0.10).

## Discussion

Environmental hyperthermia is a common issue in animal agriculture that compromises animal welfare and profitability (Renaudeau et al., 2012; Baumgard and Rhoads, 2013). During HS, physiological, metabolic, and endocrine adjustments are implemented to navigate the insult; therefore, productivity is compromised as nutrients are partitioned away from anabolic processes (i.e., growth, milk production). Although the etiology of the aforementioned alterations is likely multifactorial, many of the negative consequences of HS on animal health and productivity appear mediated by decreased intestinal barrier integrity (Hall et al., 2001; Lambert, 2004; Baumgard and Rhoads, 2013). There are likely multiple reasons for how HS disturbs the GIT epithelium (Mayorga et al., 2020), but ischemia has traditionally been thought to be the main culprit. During HS, blood is redistributed from the splanchnic bed to the skin in an attempt to increase heat dissipation. This coordinated circulatory event is accompanied by reduced blood delivery to the GIT and subsequent oxidative stress (Hall et al., 1994, 2001). The ensuing epithelial oxidative damage presumably results in impaired intestinal barrier integrity, allowing the translocation of bacterial components into circulation and increasing the risk of a local and systemic inflammatory response (Hall et al., 2001; Lambert, 2009).

Given the ostensible role of oxidative stress in the etiology of HS-induced intestinal dysfunction, the use of dietary antioxidants aimed to mitigate this pathology is of particular interest. Herein, we evaluated MitoQ as a therapeutic strategy to reduce oxidative stress and ameliorate systemic inflammation during a heat load. This orally available agent localizes and accumulates within the mitochondria, thus improving its targeted antioxidant efficacy (Murphy and Smith, 2007). Administering MitoQ reduces oxidative stress and tissue injury in various disease models (Lowes et al., 2008; Supinski et al., 2009; Zhang et al., 2020); however, its role during HS remains unexplored. Therefore, study objectives were to assess the effects of MitoQ on metabolism and inflammation during an acute HS challenge.

Relative to TN pigs, the thermal load caused a substantial increase in all body temperature indices, including *T*_R_, *T*_S_, and RR, indicating that the current intense HS protocol was successfully implemented. Although MitoQ administration did not influence *T*_R_ or *T*_S_, RR in HSMitoQ pigs was slightly but significantly decreased relative to their HSCon counterparts; however, the biological importance of this small difference remains unclear, particularly as *T*_R_ and *T*_S_ were similar between heat-stressed groups.

As anticipated, HS pigs had a severe decrease in FI and marked BW loss relative to their TN counterparts. Reduced FI is a characteristic response to a heat load across species, and it likely represents a survival strategy to minimize metabolic heat production (Baumgard and Rhoads, 2013). Interestingly, the reduction in FI was less severe in HSMitoQ pigs compared to HSCon (1.5 kg vs. 0.9 kg, respectively), and this corresponded with a similar response in BW, where HSMitoQ pigs did not lose as much as their HSCon counterparts (−3.9 kg vs. −5.5 kg, respectively). It is unclear why MitoQ would have had such large effects on FI and BW during HS, especially considering that MitoQ reduced appetite and decreased the expression of orexigenic peptides in a rodent model of metabolic syndrome (Fink et al., 2017). However, if repeatable, this observation has practical implications for animal agriculture as improved growth during HS would have an impact on farm profitability.

**Figure 2.**
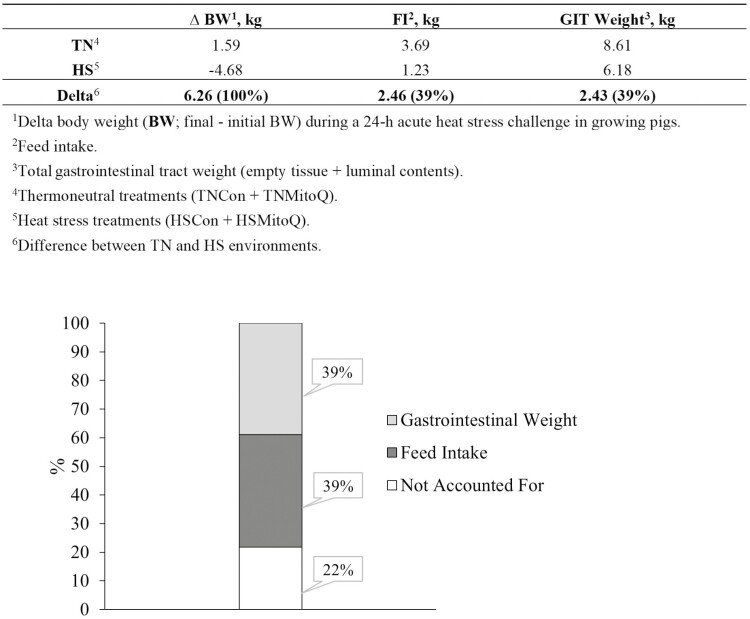
Effects of HS on BW loss during a 24 h acute heat load in growing pigs. The impact of reduced FI and decreased gastrointestinal weight was calculated and illustrated as a percentage of total BW loss.

Although decreased FI can partially explain the observed BW loss, it only accounted for ~40% of the net BW loss, suggesting additional mechanisms contribute to this altered phenotype during HS. Chronic HS exposure reduces the splanchnic bed mass (i.e., liver, GIT; Rinaldo and Le Dividich, 1991; Johnson et al., 2015); however, it is not clear if a severe and acute HS has similar effects. We have previously reported a comparable magnitude of FI and BW reduction after an acute HS challenge; however, that study was limited by the lack of GIT measurements (Mayorga et al., 2021a). Thus, to better understand this phenomenon, the current study investigated the contribution of reduced GIT mass. Overall, HS pigs had reduced GIT weight relative to their TN counterparts, and this was mainly explained by a decrease in both luminal contents and GIT tissue mass. Interestingly, HSMitoQ pigs had increased luminal contents relative to their HSCon counterparts, coinciding with the increased FI. Nevertheless, considering the total BW reduction during HS (6.26 kg) and subtracting the decrease in both FI (2.46 kg) and total GIT weight (2.43 kg), there remains a 1.4 kg difference that is not being accounted for in the net BW loss (Figure 2).

In addition to changes in GIT mass, thermoregulatory mechanisms implemented during HS (i.e., sweating, panting) can result in dehydration and thus contribute to BW loss (Akerman et al., 2016). However, hematocrit concentrations in HS pigs were comparable to the TN controls, suggesting dehydration did not meaningfully contribute to the BW loss. Another mechanism that could have affected BW is the physiological stress response to the heat. Accordingly, the corticotropin-releasing factor (**CRF**) system and other mediators of the stress response (e.g., catabolic hormones) have been implicated in the anorexia and BW loss observed in various models of chronic stress (Santos et al., 2000), but whether this holds true in our current acute HS model is unknown. Thus, understanding the mechanisms and contributions to this considerable loss of body mass is critical to developing therapeutic strategies to minimize production losses and improve animal health during environmental hyperthermia.

Interestingly, the CRF system appears to regulate gut motility by delaying gastric emptying and increasing colonic transit (Czimmer and Tache, 2017), which coincides with our anecdotal evidence that HS pigs had more stomach and less intestinal contents relative to their TN counterparts. Given these observations, we measured luminal contents (absolute and as a percentage of FI) and detected an increased proportion (a mixture of feed and water) in pigs exposed to the heat load despite reduced FI. This suggests HS altered gut peristalsis, presumably by reducing GIT motility; however, whether this is specific to the proximal or distal regions of the GIT remains to be elucidated.

Environmental hyperthermia causes a distinctive shift in carbohydrate metabolism (Baumgard and Rhoads, 2013). For instance, decreased circulating glucose occurs in various species, including rodents, pigs, and cows (Mitev et al., 2005; Sanz Fernandez et al., 2015a, b). In the current study, we observed a decrease in glucose concentrations that was only evident in HSCon pigs, while in the HSMitoQ treatment, it remained similar to their TN counterparts. This resulted in increased circulating glucose (15%) in HSMitoQ relative to HSCon pigs. A reciprocal relationship was also detected in NEFA (see below), and differences in both energetic metabolites between HS groups may be (at least partially) explained by differences in FI, as HSMitoQ pigs consumed more feed (and thus had more carbohydrates to absorb) than their HSCon counterparts.

Coinciding with inappetence, circulating insulin decreased in HS. When insulin was analyzed based on FI, heat-stressed pigs tended to have increased circulating insulin per kg of feed consumed compared to TN controls. Although this observation agrees with others (Pearce et al., 2013; Sanz Fernandez et al., 2015b), the energetics of this response seem paradoxical, especially considering that insulin is a potent anabolic hormone, yet heat-stressed animals were in a catabolic state (i.e., severe hypophagia and acute BW loss). Why heat-stressed animals have more circulating insulin than expected is not clear. HS reduces GIT barrier function, and this has been firmly established in multiple species (Hall et al., 2001; Pearce et al., 2014; Gabler et al., 2018), a scenario that allows LPS (and likely a plethora of antigens) to infiltrate into circulation. Interestingly, administering LPS induces a distinct increase in circulating insulin in various species (Waldron et al., 2006; Kvidera et al., 2017a, b); an endocrine strategy to ensure copious glucose uptake by insulin-responsive leukocytes (Horst et al., 2021), particularly as insulin sensitivity in skeletal muscle appears blunted (Ganesan et al., 2018). Ergo, the increased insulin dynamics during HS is ostensibly a strategy to partition nutrients and energy towards the immune system.

Along with altered glucose and insulin dynamics, HS has large effects on lipid metabolism, especially evident when heat-stressed animals are compared to pair-fed TN controls (Baumgard and Rhoads, 2013; Mayorga et al., 2021b). In the present study, circulating NEFA concentrations in HSMitoQ pigs were comparable to those in TN pigs, regardless of the dissimilar FI and pronounced BW loss between the two environments. In contrast, HSCon pigs had a slight increase in circulating NEFA relative to both TN and HSMitoQ pigs. Why MitoQ appeared to reduce adipose mobilization during HS is not clear. Altered lipid metabolism during HS is usually attributed to the concomitant increase in circulating insulin (a potent antilipolytic ligand); however, heat-stressed animals were hypoinsulinemic and the insulin:FI ratio did not differ between the HS treatments, suggesting other mechanisms are responsible for this conflicting result. While the extent of increased NEFA levels in HSCon pigs is minimal relative to that observed in underfed animals (Govoni et al., 2005), the mechanisms behind this distinctive NEFA pattern between the two HS treatments warrant further investigation.

Acute HS alters intestinal architecture and permeability in various models (Lambert et al., 2002; Pearce et al., 2014; Nanto-Hara et al., 2020). Intestinal dysfunction during HS and the subsequent translocation of LPS from the intestinal lumen through the basolateral membrane activates the immune system and stimulates an inflammatory response (Lambert, 2009; Koch et al., 2019). Interestingly, these changes appear to occur early during the initiation of the heat insult, and this demonstrates the GIT’s susceptibility and sensitivity to HS (Lambert et al., 2002; Pearce et al., 2014, 2015). Therefore, we evaluated white blood cell dynamics to assess the impact of the current HS protocol on stimulating an immune response. However, contrary to our expectations, we did not detect changes in circulating leukocytes in pigs exposed to the acute 24-h heat load. Although this disagrees with our previous work (Mayorga et al., 2021a), it corroborates leukocyte dynamics in various heat-stressed species (Hicks et al., 1998; Waltz et al., 2014). Utilizing changes in white blood cell counts as a marker of immune activation is complicated as their concentrations are transient and represent a combination of entry and exit from the circulating pool. Thus, it is reasonable to suggest that HS increased the recruitment of leukocytes from the circulating pool into the gut (Koch et al., 2019), and the bone marrow increased leukocyte mobilization into the blood to prevent leukopenia.

We also evaluated some aspects of the acute-phase protein response, an important mediator of the innate immune system to stress and inflammation. Previous studies have demonstrated increased circulating levels of different acute-phase proteins (i.e., Hp., serum amyloid A, LPS-binding protein) in response to acute and chronic HS (Gabler et al., 2018; Cui et al., 2019). Herein, circulating Hp was increased in heat-stressed pigs relative to their TN counterparts, while C-RP was similar between groups. Reasons for this discordant pattern in acute-phase proteins are not entirely clear. However, the acute phase response is mainly dependent on the inflammatory stimulus, and the temporal pattern varies across proteins such that C-RP can markedly increase within the first hours of the inflammatory insult and decrease afterward, while Hp exhibits a slower and prolonged increase in circulation (Cray et al., 2009). Thus, our blood sampling regimen may have prevented us from capturing the transient patterns. Regardless, the increased Hp and other acute-phase proteins described in the aforementioned HS experiments presumably stem from GIT barrier dysfunction and provide more evidence that HS is an immune-stimulating stressor.

Contrary to our hypothesis, administering MitoQ did not affect C-RP or Hp. This was surprising because previous studies have reported beneficial MitoQ effects in various inflammatory models. For instance, MitoQ treatment reduced ROS formation and decreased the proinflammatory cytokine response in an in vitro model of sepsis (Lowes et al., 2008). This was confirmed in vivo as MitoQ reduced mitochondria damage, protected against liver and renal dysfunction, and decreased the inflammatory response in a rodent model of acute sepsis (Lowes et al., 2008). Further, MitoQ attenuated gut injury and mucosal barrier dysfunction in response to intestinal ischemia-reperfusion in rodents (Hu et al., 2018). A similar observation was reported by Zhang et al. (2020), where MitoQ treatment prevented intestinal oxidative stress and inflammation and modulated mucosal barrier dysfunction following an LPS challenge. Notably, the previous studies employed different species and a dissimilar dose regimen (~4 mg/kg) than that utilized herein, which may explain the discrepancies in MitoQ effects across studies. Surprisingly, we did not detect an oxidative stress response in skeletal muscle in these exact pigs (Rudolph et al., 2021), and we did not measure oxidative stress in the intestinal tissues or circulation. Thus, it is possible that oxidative stress did not occur in our model using barrows, a scenario that would have limited our ability to test the antioxidative effects of MitoQ; though may rise questions about the potential role of biological sex on HS-mediated outcomes.

## Conclusion

Acute HS exposure caused a marked increase in all body temperature indices and a significant reduction in FI and BW. Pigs exposed to the heat load had a reduction in the GIT mass, but reduced FI coupled with decreased GIT mass did not fully explain the severe BW loss, suggesting other mechanisms contribute to this catabolic condition. Further, circulating Hp increased in response to HS, implying the current thermal load induced an acute phase response, likely due to reduced intestinal barrier integrity. Interestingly, MitoQ appeared to ameliorate the decrease in FI and the BW loss during the thermal challenge and altered some aspects of metabolism, but contrary to our expectations, no effects on inflammatory biomarkers were detected due to MitoQ treatment. Although this therapeutic approach appears to positively influence growth during HS, further research is needed to confirm the replicability of this impressive response, especially considering the lack of effects on other parameters evaluated herein.

## Abbreviations

bpm: breaths per minute
BUN: blood urea nitrogen
BW: body weight
C-RP: C-reactive protein
Con: control
CRF: corticotrophin releasing factor
FI: feed intake
GIT: gastrointestinal tract
HS: heat stress
HSCon: heat stress + control
HSMitoQ: heat stress + mitoquinol
Hp: haptoglobin
LPS: lipopolysaccharide
MitoQ: mitoquinol
NEFA: non-esterified fatty acids
P: period
ROS: reactive oxygen species
RR: respiration rate
TN: thermoneutral
TNCon: thermoneutral + control
TNMitoQ: thermoneutral + mitoquinol
T_R_: rectal temperature
T_S_: skin temperature
